# Editorial: Trauma, attachment and culture

**DOI:** 10.3389/fpsyg.2025.1688970

**Published:** 2025-09-05

**Authors:** Pia Tohme, Ian Grey, Rudy Abi-Habib

**Affiliations:** ^1^Department of Psychology and Education, School of Arts and Sciences, Lebanese American University (LAU), Beirut, Lebanon; ^2^Department of Cognitive Sciences, College of Humanities and Social Science, United Arab Emirates University (UAEU), Al Ain, United Arab Emirates

**Keywords:** trauma, attachment, culture, PTSD, intergenerational trauma, psychotherapy outcomes

Although attachment is considered to be a universal human phenomenon, it is increasingly recognized that cultural norms and practices significantly influence its development and manifestation. Attachment styles have been found to play a pivotal role in predicting psychological adjustment across the lifespan, the quality of interpersonal relationships, emotion regulation, and coping responses to stress and trauma. Through this Research Topic—*Trauma, Attachment, and Culture*—the editors bring together five distinct, yet complementary, scholarly articles that explore in various contexts, how trauma management is influenced by attachment styles which in turn are influenced by dimensions of culture. One such style, secure attachment, is most likely to facilitate effective culturally attuned processing of emotionally loaded traumatic experiences. Collectively, the research findings presented as part of this special topic serve to illustrate the multilayered interplay between traumatic experiences, attachment styles, and cultural environments across different stages of life.

The first article by Maciel et al. explores changes in attachment dimensions and behaviors amongst Brazilian women undergoing psychological or psychopharmacological interventions for posttraumatic stress disorder (PTSD) following sexual assault. Over the course of a 14-week clinical trial comparing interpersonal psychotherapy (IPT) and anti-depressant medication (sertraline), the authors describe how indices of attachment anxiety and attachment avoidance changed over the duration of the study. Interestingly, the findings highlighted that while PTSD symptoms decreased in both study groups (therapy and medication groups), attachment related avoidance increased over time, particularly among younger, non-white participants. This finding, in particular, challenges the more intuitive assumption that symptom reduction is positively correlated with attachment security. The findings suggest the possible influence of Brazil's sociopolitical context, characterized by high rates of gender-based violence in the Black and mixed-race population, on the interplay between changes in attachment and traumatic symptomatology. In other words, in Brazil, the high prevalence of violence against women may necessitate the adoption of attachment avoidance strategies as an adaptive response. This study also contributes to a growing body of work emphasizing that trauma interventions should consider attachment as a dynamic, culturally embedded variable, rather than as a static trait responsive only to symptom reduction.

The second paper by Diab and Al-Azzeh advocates for trauma-informed, survivor-centered research approaches when working with displaced communities affected by gender-based violence (GBV). The authors emphasize the need for compassionate and empowering methodologies, prioritizing the emotional and physical safety of survivors, fostering trust and reducing re-traumatization risks. They argue it is crucial to consider the challenges of power asymmetries, stigma, and societal norms that inhibit survivor disclosure in marginalized groups. The authors support the centrality of communal support and collective healing, especially for women navigating the compounded trauma of displacement, violence, and socioeconomic instability, which tend to lead to increased isolation and further trauma. In sum, this review advocates for the research process itself to be a healing space and addresses the danger of insensitive research approaches as an avenue toward re-traumatization. Research must actively create a safe space by promoting community ownership of research, thus increasing the likelihood of sustained culturally sensitive interventions.

Expanding the clinical lens, in a third contribution, Smits et al. present an in-depth exploration of Trauma-Focused Mentalization-Based Treatment (MBT-TF) for individuals with complex trauma and co-occurring personality disorders. Grounded in mentalization theory and the influence of broken epistemic trust and reduced capacity to mentalize following trauma, the study examined a group intervention lasting 6–12 months, which included psychoeducation, trauma processing, and integration. Participants shared narratives, engaged in reflective dialogue, and gradually rebuilt coherent self-other representations, based on trauma processing in a group setting which decreased avoidance and isolation. The authors also explore the role of embodied mentalization, connecting physical sensations with emotional meaning, considered to be critical in establishing safety and epistemic trust. This is illustrated through the case study of Ellen, which describes the use of MBT-TF in facilitating the reconciliation of a traumatic religious upbringing, to foster relational resilience and compassionate self-understanding. The authors support the use of this approach as a tool to confront the challenges of comorbid dissociation, stigma, and diagnostic complexity often associated with histories of interpersonal trauma.

From a developmental and cultural perspective, Al-Azzeh and Diab review the psychological impacts of maternal migration on “left-behind” children. The feminization of global labor-based migration has led to significant changes in caregiving practices, particularly in low- and middle-income countries. This review indicates that children separated from their mothers, more so than from fathers, report higher levels of loneliness, anxiety, and emotional dysregulation. However, such outcomes are mitigated by the stability and quality of substitute caregivers, cultural norms around communal caregiving, and societal attitudes toward maternal roles. In some African and Asian contexts, communal child-rearing practices can provide emotional support, despite parental absence, though care arrangements can also lead to attachment insecurity. The authors call for culturally sensitive interventions that support, not only the children, but also involve the broader caregiving ecosystems disrupted by migration. In other words, attachment is not solely dyadic bound, rather it is socially and culturally influenced.

Finally, Tortora and Schechter provide a compelling case-based contribution employing nonverbal video microanalysis to examine maternal reflective functioning during Clinician-Assisted Videofeedback Exposure Sessions (CAVES) with toddlers. Using the Dyadic Attachment-based Nonverbal Communicative Expressions (DANCE) tool, their findings reveal how mothers who have suffered from trauma often misinterpret their children's emotional signals, responding with defensive or avoidant behaviors. Through the use of CAVES, these mothers gradually shift toward greater attunement and embodied empathy. Using case studies, the study illustrates how maternal trauma history shapes not only narrative content but also nonverbal interaction patterns that underpin early attachment formation. The authors advocate for integrating body-focused, video-supported interventions in trauma-informed parenting programs. Overall, they offer a compelling argument for the therapeutic potential of nonverbal communication as a portal into emotional regulation and intergenerational repair.

In summary, the five contributions for this Research Topic provide an understanding of trauma as having an impact, not merely at the individual level, but also as a relational break embedded in cultural, structural, and interpersonal contexts. Whether through randomized trials, video analysis, research frameworks, or narrative casework, each manuscript situates attachment within broader systems, be it of race, gender, caregiving, migration, or collective meaning-making. These works challenge simplistic notions of “recovery,” and instead, offer more nuanced insights into the complex, nonlinear processes of the working through of emotions resulting from traumatic events. This can only be achieved by using diverse methodologies, ranging from quantitative trials to narrative reviews and video-based microanalysis, capturing both general and case-specific findings. These studies point to a growing recognition that trauma research must be integrative, capable of capturing not only symptoms but also triggers, avoidance, and narratives influenced by cultural contexts.

In conclusion, this Research Topic extends the Western conversation on trauma and attachment, by emphasizing the dimension of culture, as both a context of vulnerability, and a resource for resilience. It invites future scholars to explore how working through traumatic experiences emerges not only from individual insight, but from restored relationships, reflective dialogue, and culturally rooted values of the community.

